# Keeping It Simple—Pain Science Education for Patients with Chronic Pain Referred to Community-Based Rehabilitation: Translation, Adaptation, and Clinical Feasibility Testing of PNE4Adults

**DOI:** 10.3390/jcm14030771

**Published:** 2025-01-24

**Authors:** Bettina Eiger, Michael Skovdal Rathleff, Kelly Ickmans, Emma Rheel, Christian Lund Straszek

**Affiliations:** 1 Department of Health Science and Technology, Aalborg University, 9260 Gistrup, Denmark; misr@hst.aau.dk (M.S.R.); cst@dcm.aau.dk (C.L.S.); 2 Rehabilitation Center, Køge Municipality, Rådhusstræde 10C, 4600 Køge, Denmark; 3 Center for General Practice at Aalborg University, Aalborg University, 9260 Gistrup, Denmark; 4 Pain in Motion Research Group (PAIN), Department of Physiotherapy, Human Physiology and Anatomy, Faculty of Physical Education & Physiotherapy, Vrije Universiteit Brussel, Laarbeeklaan 103, 1090 Brussels, Belgium; emma.rheel@vub.be; 5 Department of Physical Medicine and Physiotherapy, Universitair Ziekenhuis Brussel, Laarbeeklaan 101, 1090 Brussels, Belgium; 6 Research Foundation–Flanders, HOEK 38, Leuvenseweg 38, 1000 Brussels, Belgium; 7 Department of Physiotherapy, University College of Northern Denmark, 9220 Aalborg, Denmark

**Keywords:** pain science education, feasibility, health literacy, PNE4Adults, chronic pain, municipality, rehabilitation, Danish

## Abstract

**Background/Objectives**: Pain science education accommodating low health literacy is needed for people with chronic pain. The purpose of this study was to translate PNE4Kids, contextually adapt it into PNE4Adults, and test the feasibility of the newly developed pain science education program (PNE4Adults) for adult patients with chronic musculoskeletal pain in the municipality. **Methods**: A three-step approach was used to (1) translate PNE4Kids into Danish, (2) adapt to age and context (PNE4Adults), and (3) test the feasibility. (1) Translation was performed by a native Dane fluent in Dutch. (2) Two think-aloud group sessions were held, with therapists and end users. (3) Feasibility was tested amongst twenty adult patients with chronic musculoskeletal pain consecutively referred for rehabilitation in the municipality. The a priori success criteria were determined to be 70% acceptability and 70% understandability. Prior to inclusion of the first patient, the study was pre-registered on clinicaltrial.gov [NCT05140031]. **Results**: Translation was successfully performed. Both the therapist and end users found the program easy to grasp, the simplicity and interactive nature of the program ingenious, and the program to be well suited to an adult population. All patients (100%), across health literacy levels, found PNE4Adults comprehensible and acceptable. **Conclusions**: The aims were successfully met. Progression to a full trial is warranted and is underway.

## 1. Introduction

According to the World Health Organization (WHO), 20–33% of the world’s population lives with painful musculoskeletal (MSK) conditions [1]. As the costs due to MSK pain correspond to almost 2% of the gross domestic products of European countries [2], these pain conditions pose a challenge for healthcare systems globally [3,4]. Patients with chronic MSK pain have high use of healthcare, reduced work ability, functional limitations in their daily lives, and diminished quality of life [5,6,7]. Current care guidelines recommend that pain science education (PSE) [8]—also called, e.g., therapeutic neuroscience education [9,10], pain neuroscience education [11,12], or explain pain [13]—is a vital part of the care delivered to individuals suffering from chronic pain [13,14,15,16,17,18,19,20]. These programs aim to reconceptualize pain and enhance biopsychosocial rehabilitation [21,22].

On a patient level, PSE has been shown to reduce patients’ pain intensity, pain catastrophizing, and fear avoidance in addition to improved physical functioning, self-efficacy, and pain knowledge [23,24,25], especially in combination with other therapies [11,26]. Combining exercise and PSE shows greater short-term improvements in pain, disability, kinesiophobia, and pain catastrophizing compared to exercise alone [16]. On a societal level, PSE has been shown to reduce health expenses [25,27]. However, some of the proposed barriers include training of the therapist delivering the education [28,29,30,31], access to training materials [32], time during consultation, and patient health literacy levels [33,34]. Despite PSE’s wide recognition in the scientific literature, implementing it in a clinical context has proven difficult [30]. Physiotherapist do not feel competent in delivering this type of biopsychosocial intervention [35,36,37] and current PSE materials may be too complex [34,38]. In Denmark, where the population is generally highly educated, nearly 4 out of 10 people face difficulties accessing, understanding, appraising, and applying health information [33]. This underlines the need to consider novel ways of delivering PSE across all levels of health literacy [32].

Recently, Pas et al. (2018) developed a Dutch PSE program to teach children with chronic pain (and their parents) about the underlying biopsychosocial mechanisms of pain, called PNE4Kids [39,40]. The PNE4Kids program consists of a manual for therapists and a board game to enhance engagement and participant involvement. It provides an easy and accessible way for the patient to grasp the concept of pain and shows promise in reducing disability and pain-related fear in children [41]. This new PSE program may also hold promise for adult patients, including those with low levels of health literacy, and enhance learning due to the practical approach. As highlighted by Wittink, H. and Oosterhaven, J., 2018, using universal health literacy precautions is recommended by multiple professional organizations for all patients, regardless of education level or health literacy [42]. Before further effectiveness testing of the program, adequate adaptation and feasibility testing in the adult population is needed, as recommended in the updated Medical Research Counsel Framework [43]. The main aim of this study was to translate and adapt the existing PNE4Kids program into an adult Danish version (“PNE4Adults”) and test the feasibility of the program among adult patients suffering from chronic MSK pain and currently undergoing community-based rehabilitation.

## 2. Materials and Methods

### 2.1. Design

**Figure 1:**
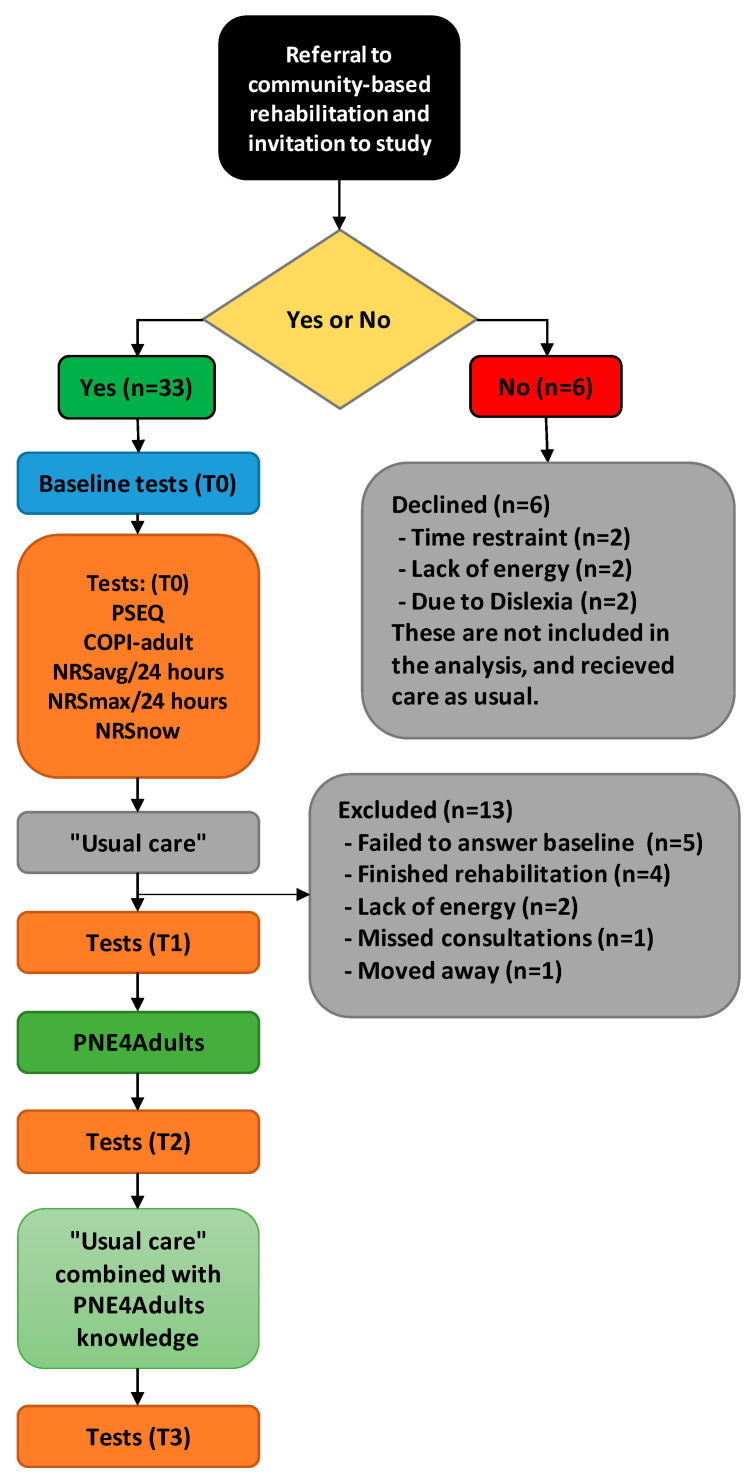
Flowchart of inclusion. ‘T0’: Timepoint 0, before first consultation, ‘T1’: Timepoint 1, after “usual care” before PNE4Adults, ‘T2’: Timepoint 2, after PNE4Adults, ‘T3’: Timepoint 3, after additional “usual care” combined with knowledge from PNE4Adults. ‘PSEQ’: Pain Self Efficacy Questionnaire, ‘COPI-Adult’: Concept Of Pain Inventory for adults, ‘NRS’: Numeric Rating Scale, ‘NRS_avg_’: The average pain in the last 24 h, ‘NRS_max_’: The maximum pain in the last 24 h, ‘NRS_now_’: The current pain, “Usual care”: routinely care received in the municipality, ‘PNE4Adults’: Pain science education.

We used a three-step approach to conduct translation, adaptation, and testing of feasibility. Step 1: translation of the PNE4Kids manual into Danish; step 2: contextual adaptation to an adult version, including two consecutive “think-aloud sessions”, one with physiotherapists and occupational therapists, and one with adult, chronic pain patients; and step 3: feasibility testing in a cross-over inspired design (Figure 1) after completion of the first two steps.

### 2.2. Setting and Context

This study was conducted at a high-volume community-based rehabilitation center in Denmark. Each year, approximately 1000 patients with MSK pain are referred for rehabilitation. Both the patients and the therapists involved in the study were from the rehabilitation center.

### 2.3. Methods

#### 2.3.1. Step 1: Translation of the PNE4Kids Program from Dutch into Danish

The original Dutch version of the PNE4Kids program was translated into Danish by a native Danish-speaking physiotherapist with no prior knowledge of pain science or the PNE4Kids program. The translator was fluent in Dutch. In an online meeting between the first author and the original authors of PNE4Kids, the new Danish version was then discussed and triangulated with the published English version for comparison of meaning. Since it was a translation of a manual of 11 pages to guide PSE rather than a questionnaire, we did not use a dual panel or forward–backward translation during this process. While psychometric testing is essential for tools like questionnaires, it is not applicable to the PNE4Adults program, which is a structured manual-based intervention. Its validity is supported by its theoretical foundation and alignment with established frameworks. The “PNE” part of the program’s name was retained for easy recognition.

#### 2.3.2. Step 2: Contextual Adaptation of PNE4Kids into PNE4Adults

We conducted a think-aloud session to adapt the PNE4Kids program into a version applicable for adult patients. We invited a group of Danish physiotherapists and occupational therapists, all healthcare professionals from the community center rehabilitation facility, with a self-reported good understanding of contemporary pain science and who had all undergone a pain science course of 48 h. See Appendix A, Table A1 for the characteristics of the participants. Prior to the think-aloud group session, the manual was forwarded to the participants to be read in advance. The think-aloud session was held in person and lasted for 1.5 h. Each part of the manual was discussed thoroughly during the session and any thoughts, uncertainties, and opinions were voiced and considered amongst the group. The session was audio-recorded and transcribed. Any suggestions for changes were talked through until consensus was reached. If consensus was not reached, the suggestion was noted and discussed within an end user group (see next section). This involved both changes with regards to the Danish adaptation but also changes related to the altered age group (i.e., the transition from children to adult patients).

After the initial think-aloud session, we used an external end user group of Danish adult patients with chronic pain to test the manual and the board game [39]. See Appendix A, Table A2 for their characteristics. The health literacy status for Panel 2 can be seen in Appendix A, Table A3, and it includes patients with low degrees of health literacy, measured with the Health Literacy Questionnaire [44]. These patients had previously attended an extensive PSE program of 10 weeks, which included pain neurophysiology and learning about the biopsychosocial aspects of pain and possibilities of self-management. The end user group did not view the manual but underwent PNE4Adults as an educational session. The group members were encouraged to voice any thoughts, questions, uncertainties, and ideas about PNE4Adults. The discussion was once again facilitated through open-end questions and was audio-recorded and subsequently transcribed.

#### 2.3.3. Step 3

##### Feasibility Study of PNE4Adults

To understand the feasibility of PNE4Adults in routine clinical practice, as well as the feasibility of obtaining answers on outcome scores, we then implemented the program during a 20-week period. We included patients who were referred to rehabilitation in the municipality suffering from chronic MSK pain, with no knowledge requirement on PSE. Prior to inclusion of the first patient, the study was pre-registered on clinicaltrial.gov [NCT05140031]. Furthermore, approval was sought by the Scientific Ethics Committee for Region North Jutland (Journal number 2021-000438), and approval was waived on 3 September 2021 for this project on the basis that it was not covered by the Committee Acts (Act no. 1338 of 01/09/2020) definition of a health sciences research project according to Danish legislation.

Eligible patients were Danish-speaking adult patients (≥18 years) with chronic MSK pain (pain persisting or recurring for ≥3 months) [45] who were cognitively able to complete the program and had no confirmed medical diagnosis indicated or related to any intellectual disorder. Recruitment was stopped when the target of 20 patients had completed the program. Twenty patients were estimated to fulfill the reasonable evaluation of the feasibility goals [46]. As part of routine practice, patients were called in for a first-time rehabilitation consultation per telephone by the secretaries. Eligible patients were informed of the project and invited to participate. If they consented, an e-link to the first battery of questionnaires was sent via secure email (e-Boks), which were filled out before the initial consultation. After informed consent from the patient was obtained, they underwent “usual rehabilitation”. Usual rehabilitation included goal setting and subsequent rehabilitation using cardio and strengthening exercises toward achieving the determined goals. After approximately 14 days, they were introduced to PNE4Adults during one or two sessions, each lasting for 45–60 min. The patients were then followed up with approximately 14 days of usual rehabilitation combined with the knowledge obtained during the PNE4Adults sessions (Figure 1). The intervention, as well as subsequent rehabilitation, were in all cases delivered by the first author (BE), who holds a Master of Pain Science and Multidisciplinary Pain Management.

##### Procedure During the Feasibility Study

Usual rehabilitation included a patient interview with individual goal setting and subsequent rehabilitation using aerobic exercises and strengthening exercises toward achieving the determined goals. We added individual PSE in the form of PNE4Adults after approximately 14 days of usual rehabilitation and subsequently continued with usual rehabilitation combined with integrating the knowledge obtained through the PNE4Adults session. The PNE4Adults session followed the developed manual (which is available at https://paininmotion.be/pne4kids (accessed on 17 May 2023) [39] and explained in brief in Appendix B) and each session lasted for 45–60 min. Firstly, the function of a normal pain system was introduced, with examples of pain being overly or under protective. The patient then taught back, giving the therapist the opportunity to evaluate the understanding and, if necessary, repeat essential key messages. Secondly, the sensitized pain system was explained. Thirdly, the person in question was asked to reflect on this new information in relation to his/her own situation and the new knowledge was subsequently integrated in the “usual rehabilitation” with any individually targeted additional measures that needed to be included, e.g., graded exposure, stress relief, graded activity, and cognitive therapies.

##### Data Collection and Outcomes for the Feasibility Study

Questionnaire-based data were collected via REDCap^®^ version 10.0.23 © 2022 Vanderbilt University and stored at a secure server at Aalborg University. Comprehensibility and acceptability were the primary feasibility outcomes. Baseline questionnaires included socio-demographic data (i.e., age, sex, marital status, work status, education level), pain duration (months), average and worst pain intensity the last 24 h, as well as current pain intensity (Numeric Rating Scale, NRS [47]). Additional baseline data included the level of symptoms related to central sensitization (Central Sensitization Inventory, CSI [48,49]), health literacy status (Health Literacy Questionnaire, HLQ [44]), pain-related knowledge (Concept of Pain, COPI-Adult [50]), pain self-efficacy (Pain Self-Efficacy Scale, PSEQ [51,52]), and pain catastrophizing (Pain Catastrophizing Scale, PCS [53]) (see specified description in Appendix B). At each of the three follow-ups (T1, T2, and T3), we included the COPI-Adult, the PSEQ, the PCS, and the three NRS measures (see Figure 1). Given that this was a feasibility study, the primary focus was on assessing acceptability and comprehensibility rather than measuring changes in outcomes. As such, no a priori hypotheses were established, nor were sample size calculations conducted for statistical testing. This aligns with the exploratory nature of the study, which prioritized gathering preliminary data to inform future research rather than testing effectiveness.

##### Comprehensibility of the PNE4Adults

The comprehensibility of PNE4Adults was measured by a participant questionnaire on a 7-point scale ranging from “very incomprehensible”, “incomprehensible”, “slightly incomprehensible”, “neither incomprehensible nor comprehensible”, “slightly comprehensible”, “comprehensible”, to “very comprehensible”. This approach only allowed us to assess if the patients found the intervention (PNE4Adults) comprehensible, and not the value of the intervention, which was clearly stated. The comprehensibility was categorized as “incomprehensible” if rated from “very incomprehensible” to “neither incomprehensible nor comprehensible” (options 1–4) and as “comprehensible” if rated from “slightly comprehensible” to “very comprehensible” (options 5–7). At least 70% of the participants should find it “comprehensible” to rate the intervention as comprehensible, which exceeded the threshold of 50% in a previous feasibility trial on understandability [54], for us to justify using a translated and adapted model initially intended for children.

##### Acceptability of the PNE4Adults

Acceptability of the combined intervention of PNE4Adults and usual care was assessed by a participant acceptability questionnaire on a 7-point scale ranging from “very unacceptable”, “unacceptable”, “slightly unacceptable”, “neither unacceptable nor acceptable”, “slightly acceptable”, “acceptable”, to “very acceptable”. This approach only allowed us to investigate if the added intervention (PNE4Adults) was acceptable to the patient, and not if the added intervention made a difference to the outcome of the rehabilitation. The acceptability was categorized as “unacceptable” if rated from “very unacceptable” to “neither unacceptable nor acceptable” (options 1–4) and as “acceptable” if rated from “slightly acceptable” to “very acceptable” (options 5–7). The intervention was rated as acceptable if at least 70% of the participants found it acceptable.

## 3. Results

### 3.1. Step 1: Translation

The translated program was discussed with the authors of the original version to clarify differences in the translation, and consensus was reached. Alterations to contextually adapt the program to an adult population included: (1) changing the example used in the first explanation of how the nociceptive system works in case of injury, (2) referring to a real-life example (in these cases, a famous cyclist who won a Tour de France stage with a broken collarbone and a case with a worker who stepped on a nail), and (3) changing the age of the case example in the final part of the program to match the adult population. Consensus was reached on all accounts.

### 3.2. Step 2: Contextual Adaptation

#### 3.2.1. Think-Aloud Session with Therapists

All participants were overall positive toward the PNE4Adults program (*n* = 6) (see Appendix A). Further, all agreed that the visual and interactive presentation and possibility of teach-back would most likely enhance learning. Also, having the board game rather than having to depend on individual drawing skills was much preferred. There was consensus that a good understanding from the therapist presenting the program was needed to present it in a clear and concise manner. One person expressed concerns that the simplicity of the program would be too childish for some adults; however, the majority disagreed with this notion, claiming that this would depend highly on individual preference. All participants approved of the alterations that were made to adapt the program to an adult population. The military as a metaphor was thoroughly discussed as some did not understand the hierarchy of the military, which confused their understanding of the metaphor. However, consensus was reached that this metaphor worked well. In Danish, the word “defense” can be used synonymously for the military, and it was agreed that this word should be presented to the patient group, since this aligns with the positive purpose of the pain system.

#### 3.2.2. Think-Aloud Session with End Users

The patients from the end user group found the program easy to understand and not childish (*n* = 5) (see Appendix A). They valued the visual and interactive nature of the program and two of the patients (with low levels of health literacy) said that they really understood pain neurophysiology for the first time after it being presented in the form of PNE4Adults. The program generated reflections on the patients’ own pain experiences and behaviors, and they emphasized that the understanding gave the patients a big responsibility for managing their own pain. Further, the patients agreed that the Danish term “defense” for military held a more positive and protective meaning to them compared to “military”. All patients found the program comprehensible, relevant, and acceptable, including those with low degrees of health literacy.

### 3.3. Step 3: Feasibility Study

From 11 November 2021 to 22 March 2022, 39 patients were referred to the current study. Six patients declined to participate due to time restraints (*n* = 2), lack of energy (*n* = 2), and because they felt their dyslexia prevented them from adequately answering the questionnaires (*n* = 2), despite being offered the possibility of assistance reading them, and are not part of the analysis. As seen in Figure 1, the remaining 33 patients answered the questionnaire the first time, but 13 were excluded because they did not complete the questionnaire before the first consultation (due to the short time frame, not lack of interest) (*n* = 5); discontinued their rehabilitation before undergoing the PNE4Adults program (because their goals had already been met) (*n* = 4); moved to a new town (*n* = 1); or withdrew after the first questionnaire, stating that they did not have the energy to participate after all (*n* = 2) or had a long period of COVID-19 disease and several appointment cancellations (*n* = 1). All were excluded before undergoing the PNE4Adults program. This left 20 patients, as pre-defined, who completed the study and were included in the analysis. One patient failed to fill out the questionnaire at T1 (before PNE4Adults) and T2 (after PNE4Adults), and another failed to fill out the last questionnaire at T3 (after the final interview), because the follow-up questionnaires for these patients ended up in their SPAM folder. At each of the three follow-ups (T1, T2, and T3), the patients answered the COPI-Adult, the PSEQ, the PCS, and the three NRS measures (see Figure 1).

The baseline demographics of the participants are outlined in detail in Table 1.

Table 1 shows that the sample had a wide age range, equal gender distribution, and some variation in educational levels. In total, 45% of participants were still working, 55% of the participants had pain for more than 1 year, and 25% had co-morbidities.

The health literacy status is outlined in Table 2.

Table 2 shows that more than 50% of all included patients scored below 3.5 in Part 2 of the HLQ, indicating low levels of health literacy, being passive in their approach to healthcare, and having problems understanding any written information in relation to their health. Additionally, 25% scoring below 2.5 in Part 1 of the HLQ also indicated low levels of health literacy in the domains of actively managing their health and having difficulty engaging with healthcare providers.

Table 3 shows the results for the outcome measures for the feasibility study. All patients (100%) found the PNE4Adults intervention to be acceptable and comprehensible, hence exceeding the pre-defined success criteria of 70% for each outcome. There was no relevant difference in acceptability and comprehensibility between patients with different health literacy levels. In all three pain measures (NRS_avg_, NRS_max_, NRS_now_), decreases were observed on a group level from T0 to T3. Furthermore, increases in pain knowledge and pain self-efficacy were observed from T0 to T3 on a group level based on the COPI-Adult [50] and PSEQ [51] scores, respectively. Lastly, pain catastrophizing measured on the PCS [53] decreased on a group level from T0 to T3.

## 4. Discussion

### 4.1. Explanation of Findings

Translation into the Danish language was successful. Both the therapists and the patients with chronic pain indicated that the program gave them a sense of “finally getting it” and liked the interactive nature of it. The patients claimed that it resulted in reflections on their own situation and gave them a sense of ownership of their situation. From the results of the current feasibility study, we found that all patients found the PNE4Adults program to be acceptable and comprehensible, including those with low levels of health literacy.

### 4.2. Comparison with Previous Findings

PSE has been used in several feasibility studies across different age groups. These studies have primarily investigated the feasibility of using PSE as part of a rehabilitation program in the context of exploring the feasibility of running a definitive trial. In general, it appears that different forms of PSE are well accepted among patients with long-standing pain [16,40,54,55,56]. Feasibility studies also indicate that PSE is credible [57] and well accepted among younger patients, adults, and older adults. However, in a randomized feasibility study with adult patients (+50 years of age) suffering from osteoarthritic pain, Stanton et al. [38] observed that both clinicians and patients found that the PSE intervention (i.e., “Explain Pain”, “Protectometer”, and multimedia resources) was too complex. In a recent qualitative study by Oosterhaven et al., 2023, conducted with an interdisciplinary pain management program, they found that most participants had difficulties understanding the messages of the pain neuroscience education and could not integrate it in their daily lives. They concluded that health literacy levels most likely played a role [34]. As such, a simplified version of PSE was warranted, and this study now provides the first evidence of patient and clinician perspectives. Our findings of feasibility add to the current knowledge base and indicate that for individuals where written materials may be insufficient, the PNE4Adults program (including the board game) may be a viable alternative to solely written and verbal information and may also be relevant for people with low levels of health literacy.

### 4.3. Clinical Implications

Previous approaches to teach patients PSE have primarily been based on video resources, leaflets, and communication and knowledge transmission from a trained clinician [57]. The combined results from both the quantitative and qualitative analyses suggest that using PNE4Adults to communicate and teach adults about pain science is feasible, acceptable, and motivating for adult patients with chronic MSK pain. As one of the themes from the think-aloud sessions showed, participants had the sense of “I finally get it”, which may hold promise for patients with different levels of health literacy. It is often difficult to include the most vulnerable patients and patients with low levels of health literacy. These patients may have additional challenges and thereby be more susceptible to the PNE4Adults program compared to existing PSE programs due to a greater focus on practical learning and no requirements for reading skills. In this study, we successfully included some patients with low levels of health literacy, representative of the background population in Denmark, and there was no difference between those with low levels of health literacy and the rest in terms of finding the program acceptable and comprehensible, indicating that this mode of delivering PSE is also relevant for this population. Including the program during the usual educational sessions may add time or take time away from other activities. Future studies are needed to understand if this added time, or relocation of time away from other aspects of rehabilitation, is associated with better outcomes compared to not using PNE4Adults.

### 4.4. Limitations

The current study was conducted among patients referred to rehabilitation in the municipality. The aim was to use consecutive recruitment, but due to COVID-19 and associated complications, it was not possible to ask all consecutively referred participants. This may affect the generalizability of the findings but is unlikely to affect our conclusions on feasibility, as patients not being asked did not decline due to the content of the intervention. It is possible that PNE4Adults may not translate into other languages or cultural differences may limit its use in other countries. We acknowledge the potential for gender bias due to the all-female composition of the patient think-aloud group in Step 2. However, this group was representative of the demographics of the patients available at the time. Importantly, male perspectives were included through the think-aloud group of healthcare providers, and Step 3 ensured a 50% gender mix. Additionally, the adaptations made in Step 2 were minor and focused on refining language and presentation, with no changes specific to gender-related aspects. While this limitation should be considered, we believe it has minimal impact on the overall findings. A further limitation is that all interventions were delivered by the first author. In a subsequent randomized controlled trial, the interventions should be delivered by other physiotherapists to avoid this bias.

## 5. Conclusions

The PNE4kids program was successfully translated into Danish and adapted in terms of age and context into the PNE4Adults program through think-aloud sessions with therapists and patients with chronic MSK pain from a municipality setting. The PNE4Adults program was found to be acceptable, comprehensible, and feasible for use in a municipality setting. Further research is needed with a randomized controlled trial with a large enough sample size to have statistical power to evaluate the effectiveness of the program.

## Figures and Tables

**Table 1:** Baseline data (*n* = 20).

Characteristic	Measurement	Mean (±SD) or Number (%), Median and [Range]
Gender	Female	10 (50%)
Age	Years	57.7 (±13.8) [25–81 years]
Marital status (*n* = 20)	Married/Co-living	18 (90%)
	In a relationship	1 (5%)
	Single	1 (5%)
Level of education	Primary school	1 (5%)
	Upper secondary school	3 (15%)
	<3 years after upper secondary school	6 (30%)
	3–5 years after upper secondary school	9 (45%)
	>5 years after upper secondary school	1 (5%)
Employment status	Employed	9 (45%)
Reason for unemployment	On sick leave due to current illness	1 (5%)
	Retired	6 (30%)
	Early retirement	1 (5%)
	Other reasons	3 (15%)
Pain duration	3–6 months	4 (20%)
	6–12 months	5 (25%)
	1–2 years	3 (15%)
	3–5 years	3 (15%)
	5–10 years	3 (15%)
	>10 years	2 (10%)
Additional illness	Yes	5 (25%)
Central Sensitization Index (CSI)	Median	33.5 [24.0–43.2]
	Sub-clinical (0–29 points)	8 (40%)
	Mild (30–39 points)	6 (30%)
	Moderate (40–49 points)	5 (25%)
	Severe (50–59 points)	1 (5%)
	Extreme (60–100 points)	0 (%)

Data are described with mean (±SD) for normally distributed data and median (min–max) for non-normally distributed data. [Range], ‘SD’: Standard Deviation, ‘min’: minimum, ‘max’: maximum.

**Table 2:** Health Literacy Questionnaire (HLQ) of participants in the feasibility study (*n* = 20).

Part 1 * Feeling understood and supported by healthcare providers: Having sufficient information to manage my health: Actively managing my health: Social support for health: Appraisal of health information: Part 2 ** 6. Ability to actively engage with healthcare providers: 7. Navigating the healthcare system: 8. Ability to find good health information: 9. Understand health information well enough to know what to do:	Mean (±SD) *n*/N [min–max] 2.83 (±0.31), 5/20 [2.0–4.0] 2.81 (±0.40), 5/20 [1.75–4.0] 2.69 (±0.36), 8/20 [1.2–4.0] 2.98 (±0.35), 3/20 [2.4–3.8] 2.56 (±0.53), 8/20 [1.2–3.8] 3.31 (±0.63), 14/20 [1.8–4.8] 3.08 (±0.53), 13/20 [1.5–5.0] 3.48 (±0.42), 11/20 [2.6–5.0] 3.39 (±0.65), 12/20 [2.0–4.8]

* Scores below 2.5 in HLQ Part 1 indicate low levels of health literacy; ** Scores below 3.5 in HLQ Part 2 indicate low levels of health literacy; ‘Mean’: Group mean score, ‘SD’: Standard deviation, ‘*n*’: number of participants with score below threshold, ‘N’: Total number of responses ‘min’: minimum, ‘max’: maximum.

**Table 3:** Outcome measures (*n* = 20).

Acceptability, *n* (%) - Very unacceptable - Unacceptable - Slightly unacceptable - Neither unacceptable nor acceptable - Slightly acceptable - Acceptable - Very acceptable	0 (0%) 0 (0%) 0 (0%) 0 (0%) 1 (5%) 6 (30%) 13 (65%)
Comprehensibility, *n* (%) - Very incomprehensible - Incomprehensible - Slightly incomprehensible - Neither incomprehensible nor comprehensible - Slightly comprehensible - Comprehensible - Very comprehensible	0 (0%) 0 (0%) 0 (0%) 0 (0%) 0 (0%) 7 (35%) 13 (65%)
Worst pain last 24 h (NRS_max_) from 0 to 10, Median (IQR) * - T0 - T1 (*n* = 19) - T2 (*n* = 19) - T3 (*n* = 19)	5 (3–5) 5 (2–6) 4 (2–7) 3 (1–7)
Average pain last 24 h (NRS_avg_) from 0 to 10, Median (IQR) * - T0 - T1(*n* = 19) - T2 (*n* = 19) - T3 (*n* = 19)	5 (3–6.75) 5 (3–6) 3 (3–7) 3 (1–7)
Current pain (NRS_now_) from 0 to 10, Median (IQR) * - T0 - T1 (*n* = 19) - T2 (*n* = 19) - T3 (*n* = 19)	5 (2.25–6.75) 4 (1–5) 3 (1–6) 2 (1–7)
Concept Of Pain Inventory, Median (IQR) * - T0 - T1 (*n* = 19) - T2 (*n* = 19) - T3 (*n* = 19)	31.5 (30–34) 30 (27–32) 33 (30–39) 37 (32–39)
Pain Self-Efficacy Questionnaire, Median (IQR) * - T0 - T1 (*n* = 19) - T2 (*n* = 19) - T3 (*n* = 19)	23.5 (19.25–35.75) 29 (21–38) 31 (20–46) 33 (24–50)
Pain Catastrophizing Scale, Median (IQR) * - T0 - T1 (*n* = 19) - T2 (*n* = 19) - T3 (*n* = 19)	21 (13–25.5) 15 (9–26) 13 (7.25–18) 15 (15–27)

* Data from all patients at T0, data from 19/20 patients are available for T1, T2, and T3. ‘T0’: Timepoint 0, before first consultation, ‘T1’: Timepoint 1, after “usual care” before PNE4Adults, ‘T2’: Timepoint 2, after PNE4Adults, Part 1 + Part 2, ‘T3’: Timepoint 3, after additional “usual care” combined with knowledge from PNE4Adults, Part 3, ‘NRS’: Numeric Rating Scale, ‘NRSavg’: Average pain in the last 24 h, ‘NRSmax’: Maximum pain in the last 24 h, ‘NRSnow’: Current pain, ‘IQR’: interquartile range.

## Data Availability

Restrictions apply to the availability of these data. Data were obtained from Køge Kommune and are available from the authors with the permission of Køge Kommune.

## Appendix A. Tables of Participant Characteristics in Think-Aloud Sessions

**Table A1:** Panel 1: Therapists knowledgeable in pain science (*n* = 6).

Characteristic	Measurement	Mean (±SD) or No. (%)
Age	Years, mean (±SD) Range	37.8 (±7.36), 31–50 years
Sex	Female	4 (66.7)
Marital status	Married, *n* (%)	6 (100)
Work experience	Years, Mean (±SD)	13.3 (±7.20)
Education	Physiotherapist	5 (83.3)
	Occupational therapist	1 (16.7)

‘SD’: Standard Deviation; ‘No’: Number.

**Table A2:** Panel 2: Patients with chronic pain (*n* = 5).

Characteristic	Measurement	Mean (±SD) or No. (%)
Age	Years, mean (±SD) Range	58.6 (±6.58), 53–70 years
Sex	Female	5 (100)
Marital status	Married, *n* (%)	5 (100)
Level of education	Primary school, *n* (%) Upper secondary school, *n* (%) Under 3 years of education after upper secondary school, *n* (%) 4–5 years of education after upper secondary school, *n* (%) A minimum 5 years of education after upper secondary school, *n* (%)	0 (0) 0 (0) 2 (40) 3 (60) 0 (0)
Work status	Working, *n* (%)	1 (20)
Pain duration	Years, Mean (±SD)	4.8 (±0.83)
Pain intensity	NRS_avg_/last 24 h, Mean (±SD), Range	5.6 (±1.81) 3–8

‘SD’: Standard Deviation; ‘No’: Number; ‘NRS’: Numeric Rating Scale, ‘NRS_avg_’: The average pain in the last 24 h.

**Table A3:** Health literacy status of Panel 2, patients with chronic pain (*n* = 5).

Part 1 * Feeling understood and supported by healthcare providers: Having sufficient information to manage my health: Actively managing my health: Social support for health: Appraisal of health information: Part 2 ** 6. Ability to actively engage with healthcare providers: 7. Navigating the healthcare system: 8. Ability to find good health information: 9. Understand health information well enough to know what to do:	Mean (±SD) *n*/N [min–max] 3.15 (±0.10), 0/5 [3–3.75] 2.95 (± 0.30), 1/5 [2.25–3.25] 3.16 (±0.42), 0/5 [2.6–3.6] 2.96 (±0.55), 2/5 [2.2–3.8] 2.76 (±0.57), 1/5 [2.4–3.2] 3.56 (±0.38), 3/5 [2.8–4.6] 3.37 (±0.58), 1/5 [3.1–3.6] 3.64 (±0.43), 2/5 [3.0–3.8] 3.96 (±0.42), 1/5 [3.2–4.6]

* Scores below 2.5 in HLQ Part 1 indicate low levels of health literacy. ** Scores below 3.5 in HLQ Part 2 indicate low levels of health literacy. ‘Mean’: Group mean score, ‘SD’: Standard deviation, ‘*n*’: number of participants with score below threshold, ‘N’: Total number of responses, ‘min’: minimum, ‘max’: maximum.

## Appendix B

### Appendix B.1. Description of PNE4Adults

#### Appendix B.1.1. Introducing the Board

The PNE4Adults “board” is fabric, with the outline of a person, and there are moveable pieces. In the head, there is a “computer room”, representing the brain, revealing space for several “computers” representing, e.g., cognition, emotions, past experiences, contextual factors, etc., and a toy soldier, “the general”, representing the thalamus. An “elevator”, representing the spinal cord, brings messages up and down through the system, and at the level of the spinal cord is another toy soldier, “the lieutenant”, representing the neurochemicals in the synaptic gap of the dorsal horn. Throughout the body are “electrical cables”, representing nerves, and at the end of them are several toy soldiers, “privates”, representing nociceptors. Small wooden squares, “danger messages”, representing nociception from the body, are moved from the periphery though the “electrical cables”, possibly past the “lieutenant”, toward the “general”, where it is determined if the body should be protected by pain. The whole system is called “the defense”, representing the pain system.

#### Appendix B.1.2. Part 1

The first part of the education is introducing a normal pain system, where nociception is experienced. Situations of up- and downregulation of the nociceptive input, resulting in increase or decrease in the pain experience are described.

#### Appendix B.1.3. Part 2

The second part of the PNE4Adults education describes changes happening within a sensitized pain system, using case examples.

#### Appendix B.1.4. Part 3

During rehabilitation, areas deemed relevant for the patient in question are incorporated. Using cognitive behavioral techniques, the therapist asks the patient to reflect on the knowledge presented during Parts 1 and 2. This could, e.g., be addressing identified fear of movement during physical rehabilitation, where the patient is asked to identify what thoughts are contributing to the fear of moving, and then subsequently addressing those, e.g., through in vivo exposure. Other examples and further clarification of the manual can be found on the website: https://paininmotion.be/pne4kids [39].

### Appendix B.2. Description of Baseline Data and Outcome Measures

#### Appendix B.2.1. Central Sensitization Inventory

Symptoms related to central sensitization (CS) were assessed with the Danish version of the Central Sensitization Inventory (CSI-DK) [58]. This self-reported questionnaire was designed to screen for health-related symptoms that are common in CS-related disorders within chronic pain patients [48]. The CSI-DK contains 25 items, with a score range of 0 to 100, describing symptoms related to CS with the following response options “never”, “rarely”, “sometimes”, “often”, or “always”. The CSI-DK is a psychometrically-sound instrument and was found to be reliable and valid for quantifying the severity of central sensitization-related symptoms [59].

#### Appendix B.2.2. Health Literacy Questionnaire

The level of health literacy describes the individual’s ability to access and use health-related information in promoting health. Health literacy was assessed using the Danish version of the Health Literacy Questionnaire (HLQ) [44], which is a patient-reported measure consisting of 44 questions covering 9 distinct areas of health literacy. Areas 1–5 are rated on a four-point ordinal scale (“strongly disagree”, “disagree”, “agree”, and “strongly agree”) and areas 6–9 are rated on a five-point options scale (“cannot do”, “very difficult”, “quite difficult”, “quite easy”, and “very easy”). The HLQ was found to be robust for assessing health literacy [44].

#### Appendix B.2.3. Concept of Pain Inventory

To assess beliefs and knowledge of pain science, a Danish version of the Concept of Pain Inventory (COPI-adult) [60] was used. The Concept of Pain Inventory was originally developed in children [61] and recently an adult version, the COPI-adult, was created [50]. It is a 13-item self-reported questionnaire with the following response options; “strongly disagree”, “disagree”, “unsure”, “agree”, and “strongly agree”. It is a unidimensional scale that has shown good test–retest reliability. Higher COPI-adult scores reflect greater alignment with contemporary pain science. Increasing knowledge of contemporary pain science has proven to improve functional status, decrease the level of catastrophizing, decrease pain intensity and health care utility, and increase self-efficacy in patients with musculoskeletal pain [26].

#### Appendix B.2.4. Pain Self-Efficacy

Pain self-efficacy (assessed by the Pain Self-Efficacy Questionnaire, PSEQ [51,52,62]), measures how confident a person feels in dealing with everyday life despite having chronic pain. The PSEQ consists of 10 items, rated on a 7-point Likert scale ranging from 0 (“not confident at all”) to 6 (“very confident”). It can be used to screen patients and as an outcome measure [62]. The Danish version of the PSEQ showed acceptable psychometric properties with high reliability in patients with chronic pain [52].

#### Appendix B.2.5. Pain Intensity

Pain intensity was assessed on Numeric Rating Scale (NRS) [47] with answer options ranging from 0 = (“no pain”) to 10 = (“worst imaginable pain”). Pain intensity was assessed as average pain intensity during the preceding 24 h (NRS_avg_), worst pain intensity during the preceding 24 h (NRS_max_), and current pain intensity (NRS_now_), as they all seem to convey different aspects of pain [47,63] and together provide a more accurate measure of pain [64].

#### Appendix B.2.6. Pain Catastrophizing Scale

The Pain Catastrophizing Scale (PCS) [53] was used to assess catastrophizing in relation to actual or anticipated pain. In 2014, the PCS was adapted into a Danish version [65]. The questionnaire has 3 sub-scales (i.e., rumination, magnification, and helplessness) and contains a total of 13 items. Patients use the same 5-point Likert-scale to respond to all 13 items, ranging from “not at all” to “very much so”. The PCS was found to have good internal reliability and test–retest reliability for the total score [66].
